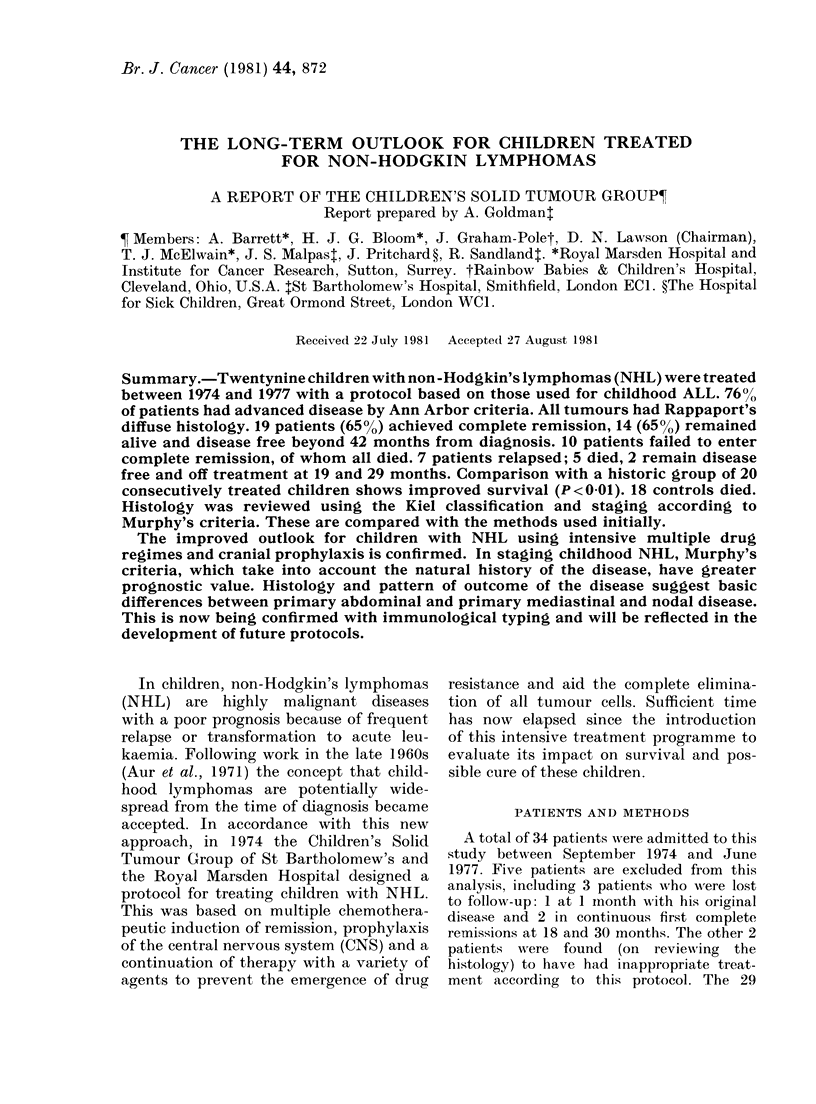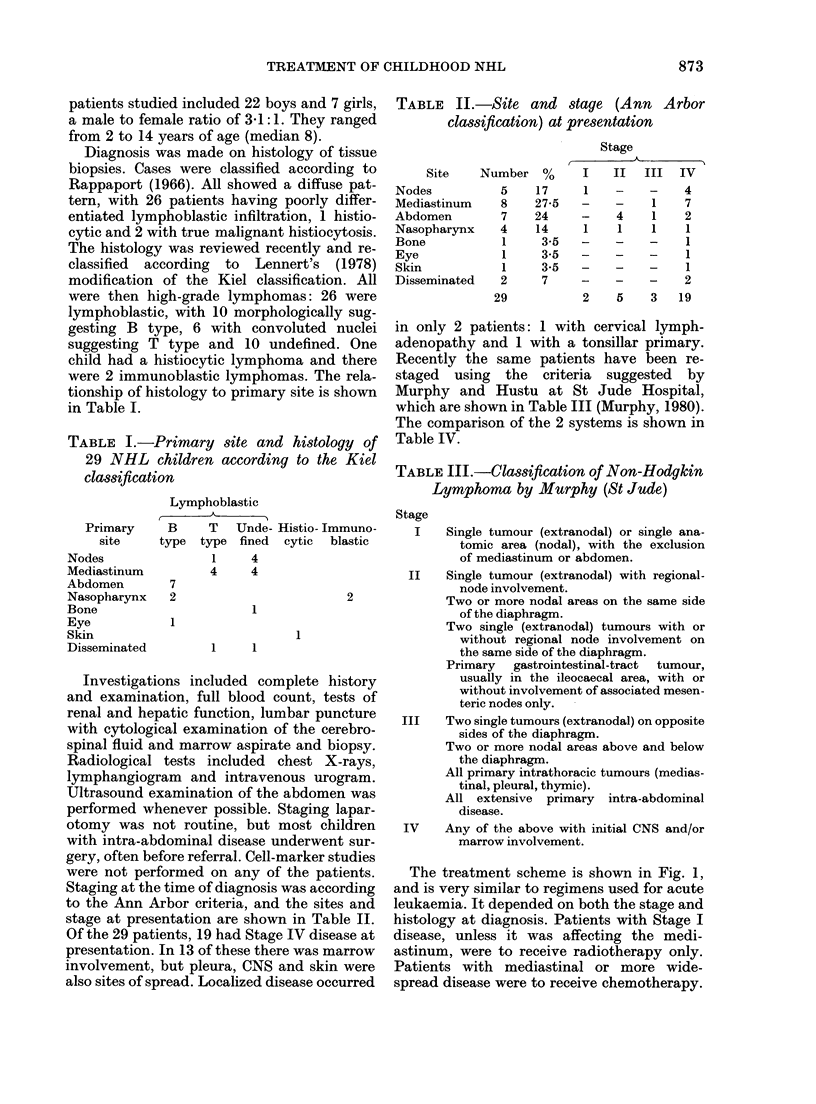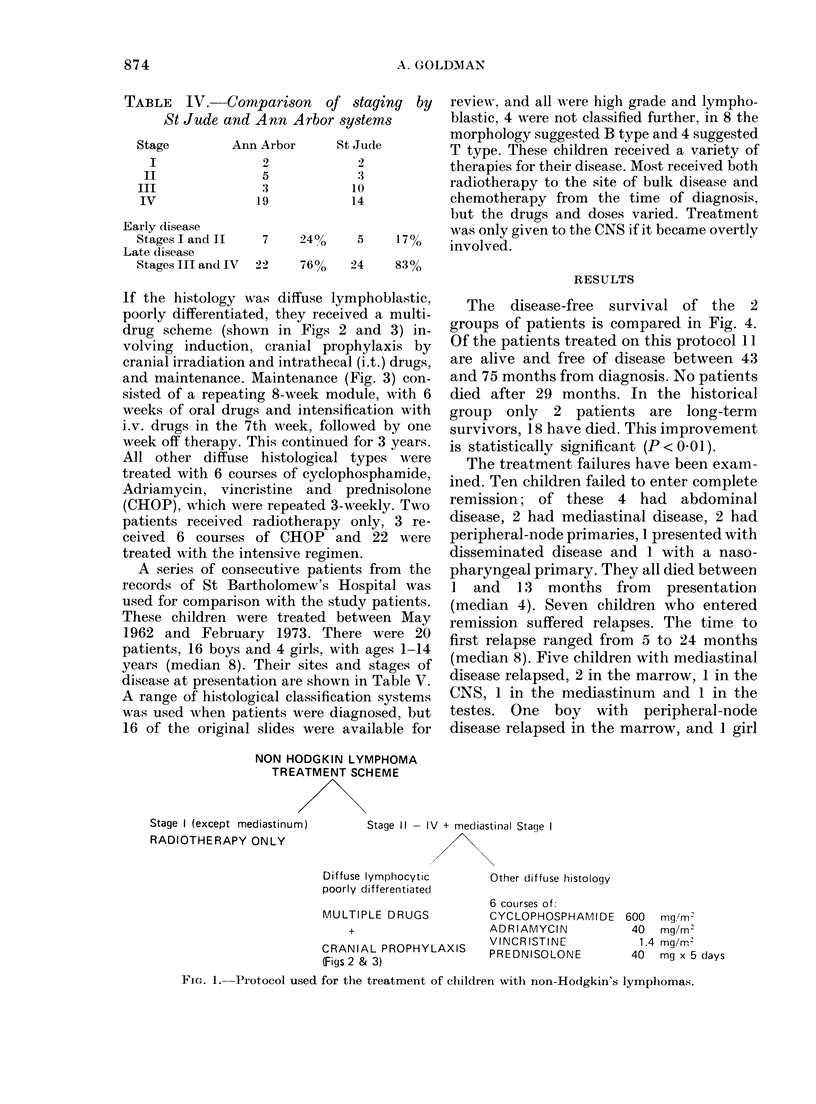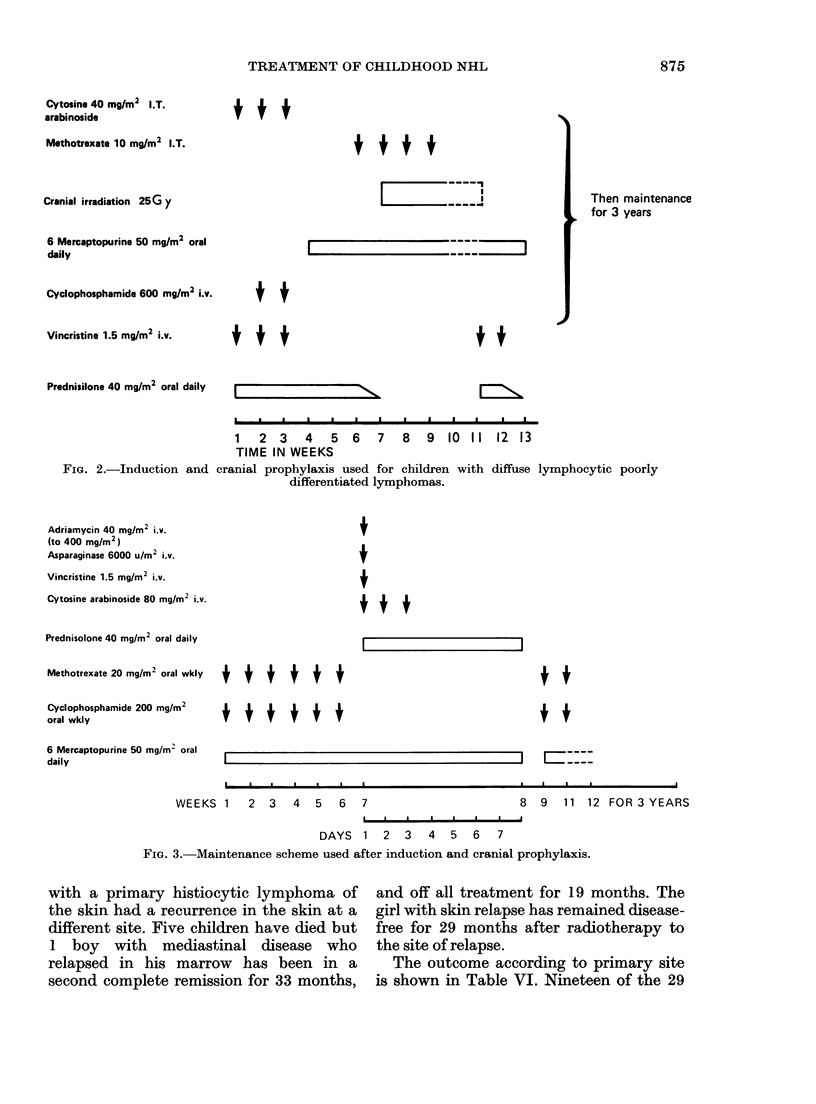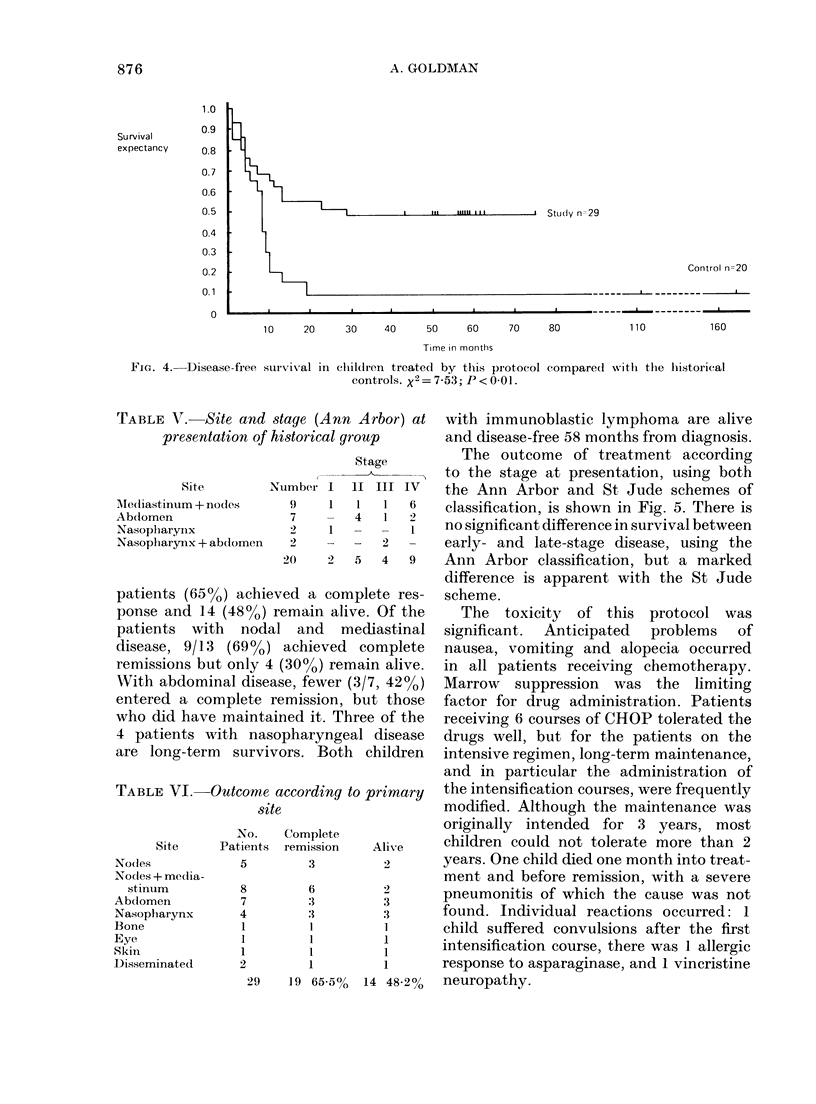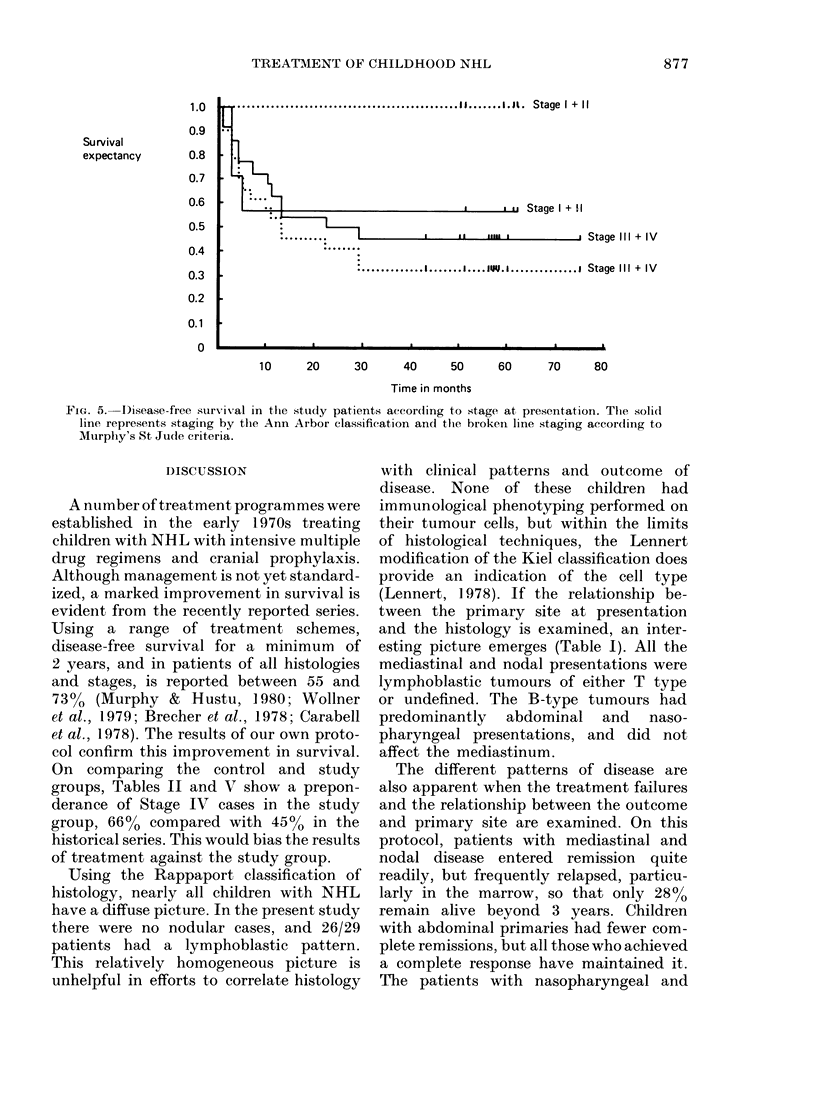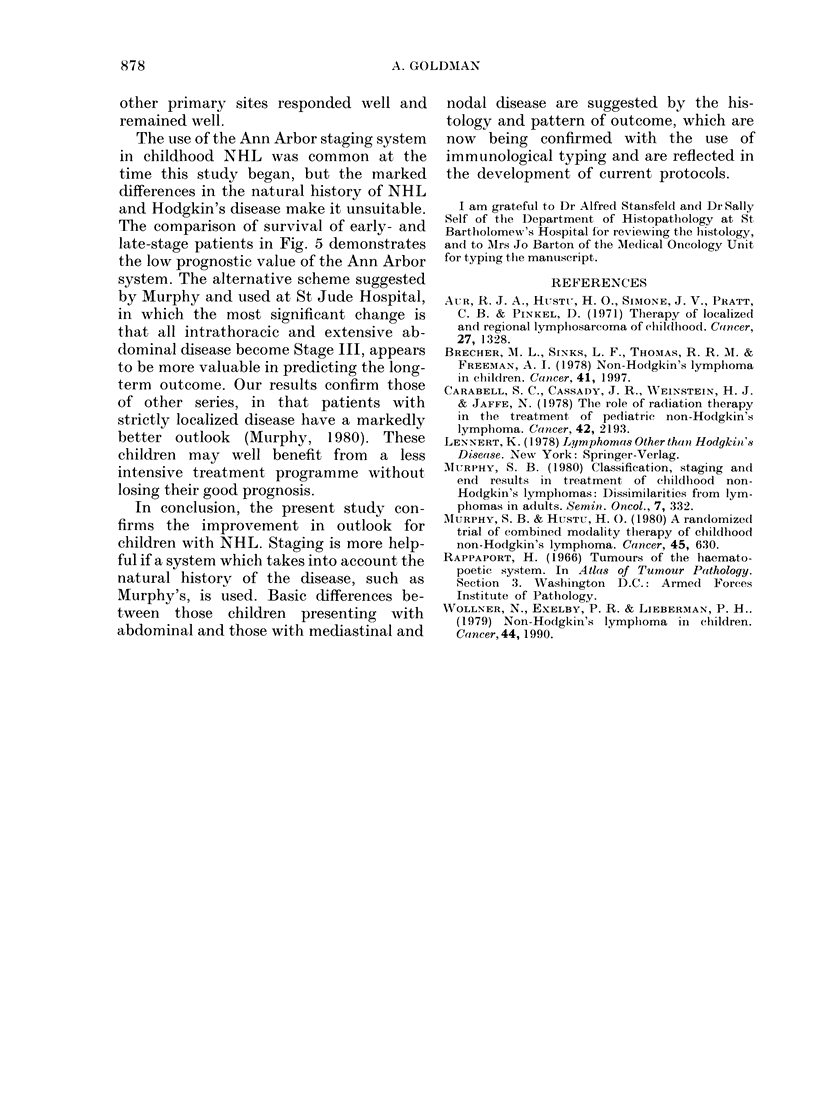# The long-term outlook for children treated for non-Hodgkin lymphomas. A report of the Children's Solid Tumour Group.

**DOI:** 10.1038/bjc.1981.286

**Published:** 1981-12

**Authors:** A. Goldman

## Abstract

Twentynine children with non-Hodgkin's lymphomas (NHL) were treated between 1974 and 1977 with a protocol based on those used for childhood ALL. 76% of patients had advanced disease by Ann Arbor criteria. All tumours had Rappaport's diffuse histology. 19 patients (65%) achieved complete remission, 14 (65%) remained alive and disease free beyond 42 months from diagnosis. 10 patients failed to enter complete remission, of whom all died. 7 patients relapsed; 5 died, 2 remain disease free and off treatment at 19 and 29 months. Comparison with a historic group of 20 consecutively treated children shows improved survival (P less than 0.01). 18 controls died. Histology was reviewed using the Kiel classification and staging according to Murphy's criteria. These are compared with the methods used initially. The improved outlook for children with NHL using intensive multiple drug regimes and cranial prophylaxis is confirmed. In staging childhood NHL, Murphy's criteria, which take into account the natural history of the disease, have greater prognosis value. Histology and pattern of outcome of the disease suggest basic differences between primary abdominal and primary mediastinal and nodal disease. This is now being confirmed with immunological typing and will be reflected in the development of future protocols.


*Br. J. Cancer* (1981) **44**, 872

### THE LONG-TERM OUTLOOK FOR CHILDREN TREATED FOR NON-HODGKIN LYMPHOMAS

A REPORT OF THE CHILDREN'S SOLID TUMOUR GROUP¶
Report prepared by A. Goldman‡

¶ Members: A. Barrett*, H. J. G. Bloom*, J. Graham-Pole†, D. N. Lawson (Chairman), T. J. McElwain*, J. S. Malpas‡, J. Pritchard§, R. Sandland‡. *Royal Marsden Hospital and Institute for Cancer Research, Sutton, Surrey. †Rainbow Babies & Children's Hospital, Cleveland, Ohio, U.S.A. ‡St Bartholomew's Hospital, Smithfield, London EC1. §The Hospital for Sick Children, Great Ormond Street, London WC1.

Received 22 July 1981 Accepted 27 August 1981

**Summary.—Twentynine children with non-Hodgkin's lymphomas (NHL) were treated between 1974 and 1977 with a protocol based on those used for childhood ALL. 76% of patients had advanced disease by Ann Arbor criteria. All tumours had Rappaport's diffuse histology. 19 patients (65%) achieved complete remission, 14 (65%) remained alive and disease free beyond 42 months from diagnosis. 10 patients failed to enter complete remission, of whom all died. 7 patients relapsed; 5 died, 2 remain disease free and off treatment at 19 and 29 months. Comparison with a historic group of 20 consecutively treated children shows improved survival ($P<0{\cdot}01$). 18 controls died. Histology was reviewed using the Kiel classification and staging according to Murphy's criteria. These are compared with the methods used initially.**

**The improved outlook for children with NHL using intensive multiple drug regimes and cranial prophylaxis is confirmed. In staging childhood NHL, Murphy's criteria, which take into account the natural history of the disease, have greater prognostic value. Histology and pattern of outcome of the disease suggest basic differences between primary abdominal and primary mediastinal and nodal disease. This is now being confirmed with immunological typing and will be reflected in the development of future protocols.**

In children, non-Hodgkin's lymphomas (NHL) are highly malignant diseases with a poor prognosis because of frequent relapse or transformation to acute leukaemia. Following work in the late 1960s (Aur *et al.*, 1971) the concept that childhood lymphomas are potentially widespread from the time of diagnosis became accepted. In accordance with this new approach, in 1974 the Children's Solid Tumour Group of St Bartholomew's and the Royal Marsden Hospital designed a protocol for treating children with NHL. This was based on multiple chemotherapeutic induction of remission, prophylaxis of the central nervous system (CNS) and a continuation of therapy with a variety of agents to prevent the emergence of drug resistance and aid the complete elimination of all tumour cells. Sufficient time has now elapsed since the introduction of this intensive treatment programme to evaluate its impact on survival and possible cure of these children.

### PATIENTS AND METHODS

A total of 34 patients were admitted to this study between September 1974 and June 1977. Five patients are excluded from this analysis, including 3 patients who were lost to follow-up: 1 at 1 month with his original disease and 2 in continuous first complete remissions at 18 and 30 months. The other 2 patients were found (on reviewing the histology) to have had inappropriate treatment according to this protocol. The 29

patients studied included 22 boys and 7 girls, a male to female ratio of 3·1:1. They ranged from 2 to 14 years of age (median 8).

Diagnosis was made on histology of tissue biopsies. Cases were classified according to Rappaport (1966). All showed a diffuse pattern, with 26 patients having poorly differentiated lymphoblastic infiltration, 1 histiocytic and 2 with true malignant histiocytosis. The histology was reviewed recently and reclassified according to Lennert's (1978) modification of the Kiel classification. All were then high-grade lymphomas: 26 were lymphoblastic, with 10 morphologically suggesting B type, 6 with convoluted nuclei suggesting T type and 10 undefined. One child had a histiocytic lymphoma and there were 2 immunoblastic lymphomas. The relationship of histology to primary site is shown in Table I.

TABLE I.—*Primary site and histology of 29 NHL children according to the Kiel classification*

| Primary site | Lymphoblastic B type | T type | Undefined | Histiocytic | Immunoblastic |
|---|---|---|---|---|---|
| Nodes | | 1 | 4 | | |
| Mediastinum | | 4 | 4 | | |
| Abdomen | 7 | | | | |
| Nasopharynx | 2 | | | | 2 |
| Bone | | | 1 | | |
| Eye | 1 | | | | |
| Skin | | | | 1 | |
| Disseminated | | 1 | 1 | | |

Investigations included complete history and examination, full blood count, tests of renal and hepatic function, lumbar puncture with cytological examination of the cerebrospinal fluid and marrow aspirate and biopsy. Radiological tests included chest X-rays, lymphangiogram and intravenous urogram. Ultrasound examination of the abdomen was performed whenever possible. Staging laparotomy was not routine, but most children with intra-abdominal disease underwent surgery, often before referral. Cell-marker studies were not performed on any of the patients. Staging at the time of diagnosis was according to the Ann Arbor criteria, and the sites and stage at presentation are shown in Table II. Of the 29 patients, 19 had Stage IV disease at presentation. In 13 of these there was marrow involvement, but pleura, CNS and skin were also sites of spread. Localized disease occurred in only 2 patients: 1 with cervical lymphadenopathy and 1 with a tonsillar primary. Recently the same patients have been restaged using the criteria suggested by Murphy and Hustu at St Jude Hospital, which are shown in Table III (Murphy, 1980). The comparison of the 2 systems is shown in Table IV.

TABLE II.—*Site and stage (Ann Arbor classification) at presentation*

| Site | Number | % | Stage I | II | III | IV |
|---|---|---|---|---|---|---|
| Nodes | 5 | 17 | 1 | – | – | 4 |
| Mediastinum | 8 | 27·5 | – | – | 1 | 7 |
| Abdomen | 7 | 24 | – | 4 | 1 | 2 |
| Nasopharynx | 4 | 14 | 1 | 1 | 1 | 1 |
| Bone | 1 | 3·5 | – | – | – | 1 |
| Eye | 1 | 3·5 | – | – | – | 1 |
| Skin | 1 | 3·5 | – | – | – | 1 |
| Disseminated | 2 | 7 | – | – | – | 2 |
| | 29 | | 2 | 5 | 3 | 19 |

TABLE III.—*Classification of Non-Hodgkin Lymphoma by Murphy (St Jude)*

| Stage | |
|---|---|
| I | Single tumour (extranodal) or single anatomic area (nodal), with the exclusion of mediastinum or abdomen. |
| II | Single tumour (extranodal) with regional-node involvement. |
| | Two or more nodal areas on the same side of the diaphragm. |
| | Two single (extranodal) tumours with or without regional node involvement on the same side of the diaphragm. |
| | Primary gastrointestinal-tract tumour, usually in the ileocaecal area, with or without involvement of associated mesenteric nodes only. |
| III | Two single tumours (extranodal) on opposite sides of the diaphragm. |
| | Two or more nodal areas above and below the diaphragm. |
| | All primary intrathoracic tumours (mediastinal, pleural, thymic). |
| | All extensive primary intra-abdominal disease. |
| IV | Any of the above with initial CNS and/or marrow involvement. |

The treatment scheme is shown in Fig. 1, and is very similar to regimens used for acute leukaemia. It depended on both the stage and histology at diagnosis. Patients with Stage I disease, unless it was affecting the mediastinum, were to receive radiotherapy only. Patients with mediastinal or more widespread disease were to receive chemotherapy.

TABLE IV.—*Comparison of staging by St Jude and Ann Arbor systems*

| Stage | Ann Arbor | | St Jude | |
|---|---|---|---|---|
| I | 2 | | 2 | |
| II | 5 | | 3 | |
| III | 3 | | 10 | |
| IV | 19 | | 14 | |
| Early disease | | | | |
| Stages I and II | 7 | 24% | 5 | 17% |
| Late disease | | | | |
| Stages III and IV | 22 | 76% | 24 | 83% |

If the histology was diffuse lymphoblastic, poorly differentiated, they received a multidrug scheme (shown in Figs 2 and 3) involving induction, cranial prophylaxis by cranial irradiation and intrathecal (i.t.) drugs, and maintenance. Maintenance (Fig. 3) consisted of a repeating 8-week module, with 6 weeks of oral drugs and intensification with i.v. drugs in the 7th week, followed by one week off therapy. This continued for 3 years. All other diffuse histological types were treated with 6 courses of cyclophosphamide, Adriamycin, vincristine and prednisolone (CHOP), which were repeated 3-weekly. Two patients received radiotherapy only, 3 received 6 courses of CHOP and 22 were treated with the intensive regimen.

A series of consecutive patients from the records of St Bartholomew's Hospital was used for comparison with the study patients. These children were treated between May 1962 and February 1973. There were 20 patients, 16 boys and 4 girls, with ages 1–14 years (median 8). Their sites and stages of disease at presentation are shown in Table V. A range of histological classification systems was used when patients were diagnosed, but 16 of the original slides were available for review, and all were high grade and lymphoblastic, 4 were not classified further, in 8 the morphology suggested B type and 4 suggested T type. These children received a variety of therapies for their disease. Most received both radiotherapy to the site of bulk disease and chemotherapy from the time of diagnosis, but the drugs and doses varied. Treatment was only given to the CNS if it became overtly involved.

### RESULTS

The disease-free survival of the 2 groups of patients is compared in Fig. 4. Of the patients treated on this protocol 11 are alive and free of disease between 43 and 75 months from diagnosis. No patients died after 29 months. In the historical group only 2 patients are long-term survivors, 18 have died. This improvement is statistically significant ($P < 0{\cdot}01$).

The treatment failures have been examined. Ten children failed to enter complete remission; of these 4 had abdominal disease, 2 had mediastinal disease, 2 had peripheral-node primaries, 1 presented with disseminated disease and 1 with a nasopharyngeal primary. They all died between 1 and 13 months from presentation (median 4). Seven children who entered remission suffered relapses. The time to first relapse ranged from 5 to 24 months (median 8). Five children with mediastinal disease relapsed, 2 in the marrow, 1 in the CNS, 1 in the mediastinum and 1 in the testes. One boy with peripheral-node disease relapsed in the marrow, and 1 girl

FIG. 1.—Protocol used for the treatment of children with non-Hodgkin's lymphomas.

FIG. 2.—Induction and cranial prophylaxis used for children with diffuse lymphocytic poorly differentiated lymphomas.

FIG. 3.—Maintenance scheme used after induction and cranial prophylaxis.

with a primary histiocytic lymphoma of the skin had a recurrence in the skin at a different site. Five children have died but 1 boy with mediastinal disease who relapsed in his marrow has been in a second complete remission for 33 months, and off all treatment for 19 months. The girl with skin relapse has remained disease-free for 29 months after radiotherapy to the site of relapse.

The outcome according to primary site is shown in Table VI. Nineteen of the 29

FIG. 4.—Disease-free survival in children treated by this protocol compared with the historical controls. $\chi^2 = 7{\cdot}53$; $P < 0{\cdot}01$.

TABLE V.—*Site and stage (Ann Arbor) at presentation of historical group*

| | | Stage | | | |
|---|---|---|---|---|---|
| Site | Number | I | II | III | IV |
| Mediastinum + nodes | 9 | 1 | 1 | 1 | 6 |
| Abdomen | 7 | – | 4 | 1 | 2 |
| Nasopharynx | 2 | 1 | – | – | 1 |
| Nasopharynx + abdomen | 2 | – | – | 2 | – |
| | 20 | 2 | 5 | 4 | 9 |

patients (65%) achieved a complete response and 14 (48%) remain alive. Of the patients with nodal and mediastinal disease, 9/13 (69%) achieved complete remissions but only 4 (30%) remain alive. With abdominal disease, fewer (3/7, 42%) entered a complete remission, but those who did have maintained it. Three of the 4 patients with nasopharyngeal disease are long-term survivors. Both children

TABLE VI.—*Outcome according to primary site*

| Site | No. Patients | Complete remission | Alive |
|---|---|---|---|
| Nodes | 5 | 3 | 2 |
| Nodes + mediastinum | 8 | 6 | 2 |
| Abdomen | 7 | 3 | 3 |
| Nasopharynx | 4 | 3 | 3 |
| Bone | 1 | 1 | 1 |
| Eye | 1 | 1 | 1 |
| Skin | 1 | 1 | 1 |
| Disseminated | 2 | 1 | 1 |
| | 29 | 19 65·5% | 14 48·2% |

with immunoblastic lymphoma are alive and disease-free 58 months from diagnosis.

The outcome of treatment according to the stage at presentation, using both the Ann Arbor and St Jude schemes of classification, is shown in Fig. 5. There is no significant difference in survival between early- and late-stage disease, using the Ann Arbor classification, but a marked difference is apparent with the St Jude scheme.

The toxicity of this protocol was significant. Anticipated problems of nausea, vomiting and alopecia occurred in all patients receiving chemotherapy. Marrow suppression was the limiting factor for drug administration. Patients receiving 6 courses of CHOP tolerated the drugs well, but for the patients on the intensive regimen, long-term maintenance, and in particular the administration of the intensification courses, were frequently modified. Although the maintenance was originally intended for 3 years, most children could not tolerate more than 2 years. One child died one month into treatment and before remission, with a severe pneumonitis of which the cause was not found. Individual reactions occurred: 1 child suffered convulsions after the first intensification course, there was 1 allergic response to asparaginase, and 1 vincristine neuropathy.

FIG. 5.—Disease-free survival in the study patients according to stage at presentation. The solid line represents staging by the Ann Arbor classification and the broken line staging according to Murphy's St Jude criteria.

### DISCUSSION

A number of treatment programmes were established in the early 1970s treating children with NHL with intensive multiple drug regimens and cranial prophylaxis. Although management is not yet standardized, a marked improvement in survival is evident from the recently reported series. Using a range of treatment schemes, disease-free survival for a minimum of 2 years, and in patients of all histologies and stages, is reported between 55 and 73% (Murphy & Hustu, 1980; Wollner *et al.*, 1979; Brecher *et al.*, 1978; Carabell *et al.*, 1978). The results of our own protocol confirm this improvement in survival. On comparing the control and study groups, Tables II and V show a preponderance of Stage IV cases in the study group, 66% compared with 45% in the historical series. This would bias the results of treatment against the study group.

Using the Rappaport classification of histology, nearly all children with NHL have a diffuse picture. In the present study there were no nodular cases, and 26/29 patients had a lymphoblastic pattern. This relatively homogeneous picture is unhelpful in efforts to correlate histology with clinical patterns and outcome of disease. None of these children had immunological phenotyping performed on their tumour cells, but within the limits of histological techniques, the Lennert modification of the Kiel classification does provide an indication of the cell type (Lennert, 1978). If the relationship between the primary site at presentation and the histology is examined, an interesting picture emerges (Table I). All the mediastinal and nodal presentations were lymphoblastic tumours of either T type or undefined. The B-type tumours had predominantly abdominal and nasopharyngeal presentations, and did not affect the mediastinum.

The different patterns of disease are also apparent when the treatment failures and the relationship between the outcome and primary site are examined. On this protocol, patients with mediastinal and nodal disease entered remission quite readily, but frequently relapsed, particularly in the marrow, so that only 28% remain alive beyond 3 years. Children with abdominal primaries had fewer complete remissions, but all those who achieved a complete response have maintained it. The patients with nasopharyngeal and other primary sites responded well and remained well.

The use of the Ann Arbor staging system in childhood NHL was common at the time this study began, but the marked differences in the natural history of NHL and Hodgkin's disease make it unsuitable. The comparison of survival of early- and late-stage patients in Fig. 5 demonstrates the low prognostic value of the Ann Arbor system. The alternative scheme suggested by Murphy and used at St Jude Hospital, in which the most significant change is that all intrathoracic and extensive abdominal disease become Stage III, appears to be more valuable in predicting the long-term outcome. Our results confirm those of other series, in that patients with strictly localized disease have a markedly better outlook (Murphy, 1980). These children may well benefit from a less intensive treatment programme without losing their good prognosis.

In conclusion, the present study confirms the improvement in outlook for children with NHL. Staging is more helpful if a system which takes into account the natural history of the disease, such as Murphy's, is used. Basic differences between those children presenting with abdominal and those with mediastinal and nodal disease are suggested by the histology and pattern of outcome, which are now being confirmed with the use of immunological typing and are reflected in the development of current protocols.

I am grateful to Dr Alfred Stansfeld and Dr Sally Self of the Department of Histopathology at St Bartholomew's Hospital for reviewing the histology, and to Mrs Jo Barton of the Medical Oncology Unit for typing the manuscript.

### REFERENCES

AUR, R. J. A., HUSTU, H. O., SIMONE, J. V., PRATT, C. B. & PINKEL, D. (1971) Therapy of localized and regional lymphosarcoma of childhood. *Cancer*, **27**, 1328.

BRECHER, M. L., SINKS, L. F., THOMAS, R. R. M. & FREEMAN, A. I. (1978) Non-Hodgkin's lymphoma in children. *Cancer*, **41**, 1997.

CARABELL, S. C., CASSADY, J. R., WEINSTEIN, H. J. & JAFFE, N. (1978) The role of radiation therapy in the treatment of pediatric non-Hodgkin's lymphoma. *Cancer*, **42**, 2193.

LENNERT, K. (1978) *Lymphomas Other than Hodgkin's Disease.* New York: Springer-Verlag.

MURPHY, S. B. (1980) Classification, staging and end results in treatment of childhood non-Hodgkin's lymphomas: Dissimilarities from lymphomas in adults. *Semin. Oncol.*, **7**, 332.

MURPHY, S. B. & HUSTU, H. O. (1980) A randomized trial of combined modality therapy of childhood non-Hodgkin's lymphoma. *Cancer*, **45**, 630.

RAPPAPORT, H. (1966) Tumours of the haematopoetic system. In *Atlas of Tumour Pathology.* Section 3. Washington D.C.: Armed Forces Institute of Pathology.

WOLLNER, N., EXELBY, P. R. & LIEBERMAN, P. H.. (1979) Non-Hodgkin's lymphoma in children. *Cancer*, **44**, 1990.